# Estimation of Sobolev embedding constant on a domain dividable into bounded convex domains

**DOI:** 10.1186/s13660-017-1571-0

**Published:** 2017-11-29

**Authors:** Makoto Mizuguchi, Kazuaki Tanaka, Kouta Sekine, Shin’ichi Oishi

**Affiliations:** 1 Faculty of Science and Engineering, Waseda University, 3-4-1, Okubo Shinjyuku-ku, Tokyo, 169-8555 Japan; 2 Faculty of Information Networking for Innovation and Design, Toyo University, 1-7-11 Akabanedai, kita-ku, Tokyo, 115-0053 Japan

**Keywords:** 46E35, Sobolev embedding constant, Hardy-Littlewood-Sobolev inequality, Young inequality

## Abstract

This paper is concerned with an explicit value of the embedding constant from $W^{1,q}(\Omega)$ to $L^{p}(\Omega)$ for a domain $\Omega\subset\mathbb{R}^{N}$ ($N\in\mathbb{N}$), where $1\leq q\leq p\leq\infty$. We previously proposed a formula for estimating the embedding constant on bounded and unbounded Lipschitz domains by estimating the norm of Stein’s extension operator. Although this formula can be applied to a domain Ω that can be divided into a finite number of Lipschitz domains, there was room for improvement in terms of accuracy. In this paper, we report that the accuracy of the embedding constant is significantly improved by restricting Ω to a domain dividable into bounded convex domains.

## Introduction

We consider the Sobolev type embedding constant $C_{p}(\Omega)$ from $W^{1,q}(\Omega)$ ($1\leq q\leq p\leq\infty$) to $L^{p}(\Omega)$. The constant $C_{p}(\Omega)$ satisfies

1 $$\begin{aligned} \biggl( \int_{\Omega} \bigl\vert u(x) \bigr\vert ^{p}\,dx \biggr)^{\frac {1}{p}}\leq C_{p}(\Omega) \biggl( \int_{\Omega} \bigl\vert u(x) \bigr\vert ^{q}\,dx+ \int_{\Omega} \bigl\vert \nabla u(x) \bigr\vert ^{q} \,dx \biggr)^{\frac{1}{q}} \end{aligned}$$

for all $u\in W^{1,q}(\Omega)$, where $\Omega\subset\mathbb {R}^{N}$ ($N\in\mathbb{N}$) is a bounded domain and $\vert x \vert =\sqrt{\sum_{j=1}^{N}x_{j}^{2}}$ for $x=(x_{1},\ldots, x_{N})\in \mathbb{R}^{N}$. Here, $L^{p}(\Omega)$ ($1\leq p<\infty$) is the functional space of the *p*th power Lebesgue integrable functions over Ω endowed with the norm $\Vert f \Vert _{L^{p}(\Omega)}:=(\int_{\Omega } \vert f(x) \vert ^{p}\,dx)^{1/p}$ for $f\in L^{p}(\Omega)$, and $L^{\infty}(\Omega)$ is the functional space of Lebesgue measurable functions over Ω endowed with the norm $\Vert f \Vert _{L^{\infty}(\Omega)}=\operatorname{ess\,sup}_{x\in\Omega } \vert f(x) \vert $ for $f\in L^{\infty}(\Omega)$. Moreover, $W^{k,p}(\Omega)$ is the *k*th order $L^{p}$-Sobolev space on Ω endowed with the norm $\Vert f \Vert _{W^{1,p}(\Omega)}=(\int_{\Omega} \vert f(x) \vert ^{p}\,dx+\int_{\Omega} \vert \nabla f(x) \vert ^{p}\,dx)^{1/p}$ for $f\in W^{1,p}(\Omega)$ if $1\leq p<\infty$ and $\Vert f \Vert _{W^{1,\infty}(\Omega)}=\operatorname{ess\,sup}_{x\in \Omega} \vert f(x) \vert +\operatorname{ess\,sup}_{x\in \Omega} \vert \nabla f(x) \vert $ for $f\in W^{1,\infty }(\Omega)$ if $p=\infty$.

Since inequality (1) has significance for studies on partial differential equations, many researchers studied this type of Sobolev inequality and an explicit value of $C_{p}(\Omega)$ (see, e.g., [1–7]) following the pioneering work by Sobolev [1]. In particular, our interest is in the applicability of this constant to verified numerical computation methods for PDEs which originate from Nakao’s [8] and Plum’s work [9]. These methods have been further developed by many researchers (see, e.g., [8–10] and the references therein).

The existence of $C_{p}(\Omega)$ for various domains Ω (e.g., domains with the cone condition, domains with the Lipschitz boundary, and the $(\varepsilon, \delta)$-domains) has been proven by constructing suitable extension operators from $W^{k,p}(\Omega)$ to $W^{k,p}(\mathbb{R}^{N})$ (see, e.g., [3–7]).

Several formulas for computing explicit values of $C_{p}(\Omega)$ have been proposed under suitable conditions. For example, the best constant in the classical Sobolev inequality on $\mathbb{R}^{N}$ was independently shown by Aubin [11] and Talenti [12]. For the case in which $N=1$ and $p=\infty$, the best constant of $C_{p}(\Omega)$ was proposed under some boundary conditions, e.g., the Dirichlet, the Neumann, and the periodic condition [13–17]. For a square domain $\Omega\subset\mathbb{R}^{2}$, a tight estimate of $C_{p}(\Omega)$ was provided in [10]. Moreover, the best constant for the embedding $W^{1,2}_{0}(\Omega )\hookrightarrow L^{p}(\Omega)$ ($p=3,4,5,6,7$) with a square domain $\Omega\subset\mathbb{R}^{2}$ was very sharply estimated in [18], where $W^{1,2}_{0}(\Omega)$ denotes the closure of $C^{\infty}_{0}(\Omega )$ in $W^{1,2}(\Omega)$. Furthermore, we have previously proposed a formula for computing an explicit value of $C_{p}(\Omega)$ for (bounded and unbounded) Lipschitz domains $\Omega\subset\mathbb{R}^{N}$ ($N\geq2$) by estimating the norm of Stein’s extension operator [19]. This formula can be applied to a domain Ω that can be divided into a finite number of Lipschitz domains $\Omega_{i}$ ($i=1,2,3,\ldots, n$) such that

2 $$\begin{aligned} \overline{\Omega}=\bigcup_{1\leq i\leq n} \overline{\Omega_{i}} \end{aligned}$$

and

3 $$\begin{aligned} \Omega_{i}\cap\Omega_{j}=\phi\quad (i\neq j), \end{aligned}$$

where *ϕ* is the empty set and Ω̅ denotes the closure of Ω (see Theorem 6.1). Although this formula is applicable to such general domains, the values computed by this formula are very large; see Section 4 for concrete values.

In this paper, we report that the accuracy of the estimation of $C_{p}(\Omega)$ is significantly improved by restricting each $\Omega _{i}$ to bounded convex domain. Since any bounded convex domain is a Lipschitz domain (see, e.g., [20]), the present class of Ω is somewhat special compared with the class treated in [19]. Nevertheless, the formulas presented in this paper still have applicability to various domains. To obtain a sharper estimation of $C_{p}(\Omega)$, we focus on the constants $D_{p}(\Omega)$ such that

4 $$\begin{aligned} \biggl( \int_{\Omega} \bigl\vert u(x)-u_{\Omega}(x) \bigr\vert ^{p}\,dx \biggr)^{\frac{1}{p}}\leq D_{p}(\Omega) \biggl( \int_{\Omega} \bigl\vert \nabla u(x) \bigr\vert ^{q} \,dx \biggr)^{\frac{1}{q}}\quad \mbox{for all } u\in W^{1,q}(\Omega). \end{aligned}$$

Here, $\vert \Omega \vert $ is the measure of Ω and $u_{\Omega}:\Omega\to\mathbb{R}$ is a constant function defined by $\Omega\ni x\mapsto u_{\Omega}(x)= \vert \Omega \vert ^{-1}\int_{\Omega}u(y)\,dy$. Inequality (4) is called the Sobolev-Poincaré inequality, and $D_{p}(\Omega)$ in (4) leads to the explicit value of $C_{p}(\Omega)$ (see Theorem 2.1). Inequality (4) has also been studied by many researchers (see, e.g., [21–24]). For example, for a John domain Ω, the existence of $D_{p}(\Omega)$ was shown while assuming that $1\leq q< N$, $p=Nq/(N-q)$ [23]. It was also shown that, when $p\neq Nq/(N-q)$, $D_{p}(\Omega)$ exists if and only if $W^{1,q}(\Omega)$ is continuously embedded into $L^{p}(\Omega)$ [24]. Moreover, there are several formulas for obtaining an explicit value of $D_{p}(\Omega)$ for one-dimensional domains Ω [25–27]. In the higher-dimensional cases, however, little is known about explicit values of $D_{p}(\Omega)$, except for some special cases (see, e.g., [28] and [29] for the cases in which $p=q=1$ and $p=q=2$, respectively).

We propose four theorems (Theorem 3.1 to 3.4) for obtaining explicit values of $D_{p}(\Omega)$ on a bounded convex domain Ω. Each theorem can be used under the corresponding conditions listed in Table 1.

**Table 1:** **The assumptions of** ***p*** **,** ***q*** **, and** ***N*** **imposed on Theorems** **3.1** **,** **3.2** **,** **3.3** **, and** **3.4**

**Theorem**	***p***	***q***	***N***
3.1	$2< p\leq\frac{2N}{N-1}$ (*N*>1), 2<*p*<∞ (*N* = 1)	$q\geq\frac{p}{p-1}$	*N* ≥ 1
3.2	$2< p\leq\frac{2N}{N-2}$ (*N*>2), 2<*p*<∞ (*N* = 2)	*q* = 2	*N* ≥ 2
3.3	$q\leq p<\frac{qN}{N-q}$ (*N*>*q*), *q* ≤ *p*<∞ (*N* = *q*)	*q* ≥ 1	*N* ≥ *q*
3.4	*p* = ∞	*q* ≥ 1	*N*<*q*

Theorems 3.1 and 3.2 are derived from the best constant in the Hardy-Littlewood-Sobolev inequality on $\mathbb{R}^{N}$. Theorems 3.3 and 3.4 are derived from the best constant in Young’s inequality on $\mathbb{R}^{N}$. The values of $D_{p}(\Omega)$ calculated by these theorems yield the explicit values of $C_{p}(\Omega)$ combined with Theorem 2.1.

The remainder of this paper is organized as follows. In Section 2, we propose Theorem 2.1 in which a formula for deriving an explicit value of $C_{p}(\Omega)$ from known $D_{p}(\Omega)$ is provided. In Section 3, we prove the four formulas (Theorems 3.1 to 3.4) for obtaining the explicit values of $D_{p}(\Omega)$. In Section 4, we present examples where explicit values of $C_{p}(\Omega)$ are estimated for certain domains.

## Estimation of embedding constant $C_{p}(\Omega)$

The following notation is used throughout this paper. For any bounded domain $S\subset\mathbb{R}^{N}$ ($N\in\mathbb{N}$), we define $d_{S}$:=$\sup_{x,y\in S} \vert x-y \vert $. The closed ball centered around $z\in\mathbb{R}^{N}$ with radius $\rho >0$ is denoted by $B(z,\rho):=\{x\in\mathbb{R}^{N}\mid \vert x-z \vert \leq\rho\}$. For $m\geq1$, let $m'$ be Hölder’s conjugate of *m*, that is, $m'$ is defined by

$$\begin{aligned} \textstyle\begin{cases} m'=\infty,& \mbox{if } m=1,\\ m'=\frac{m}{m-1},&\mbox{if } 1< m< \infty,\\ m'=1,&\mbox{if } m=\infty. \end{cases}\displaystyle \end{aligned}$$

For two domains $\Omega\subseteq\mathbb{R}^{N}$ and $\Omega'\subseteq \mathbb{R}^{N}$ such that $\Omega\subseteq\Omega'$, we define the operator $E_{\Omega,\Omega'}:L^{p}(\Omega)\to L^{p}(\Omega')$ ($1\leq p\leq\infty$) by

$$\begin{aligned} (E_{\Omega,\Omega'}f ) (x)= \textstyle\begin{cases} f(x),&x\in\Omega,\\ 0,&x\in\Omega'\setminus\Omega \end{cases}\displaystyle \end{aligned}$$

for $f\in L^{p}(\Omega)$. Note that $E_{\Omega,\Omega'}f\in L^{p}(\Omega')$ satisfies

$$\Vert E_{\Omega,\Omega'}f \Vert _{L^{p}(\Omega')}= \Vert f \Vert _{L^{p}(\Omega)}. $$

In the following theorem, we provide a formula for obtaining an explicit value of $C_{p}(\Omega)$ from known $D_{p}(\Omega)$.

### Theorem 2.1

*Let* $\Omega\subset\mathbb{R}^{N}$ ($N\in\mathbb{N}$) *be a bounded domain*, *and let* *p* *and* *q* *satisfy* $1\leq q\leq p\leq\infty$. *Suppose that there exists a finite number of bounded domains* $\Omega _{i}$ ($i=1,2,3,\ldots, n$) *satisfying* (2) *and* (3). *Moreover*, *suppose that*, *for every* $\Omega_{i}$ ($i=1,2,3,\ldots, n$), *there exist constants* $D_{p}(\Omega_{i})$ *such that*

5 $$\begin{aligned} \Vert u-u_{\Omega_{i}} \Vert _{L^{p}(\Omega_{i})}\leq D_{p}(\Omega_{i}) \Vert \nabla u \Vert _{L^{q}(\Omega_{i})}\quad \textit{for all } u\in W^{1,q}(\Omega_{i}). \end{aligned}$$

*Then* (1) *holds valid for*

6 $$\begin{aligned} C_{p}(\Omega)= \textstyle\begin{cases} \displaystyle\max \Bigl(1, \max_{1\leq i\leq n}D_{\infty}(\Omega_{i}) \Bigr) & (p=q=\infty),\\ \displaystyle2^{1-\frac{1}{q}}\max \Bigl(\max_{1\leq i\leq n} \vert \Omega _{i} \vert ^{\frac{1}{p}-\frac{1}{q}}, \max_{1\leq i\leq n}D_{p}(\Omega_{i}) \Bigr) & (\textit{otherwise}), \end{cases}\displaystyle \end{aligned}$$

*where this formula is understood with* $1/\infty=0$ *when* $p=\infty$ *and*/*or* $q=\infty$.

### Proof

Let $u\in W^{1,q}(\Omega)$. Since every $\Omega_{i}$ is bounded, Hölder’s inequality states that

7 $$\begin{aligned} \Vert u_{\Omega_{i}} \Vert _{L^{p}(\Omega_{i})}&= \biggl\vert \int_{\Omega_{i}} \vert \Omega_{i} \vert ^{-1}u(y)\,dy \biggr\vert \Vert 1 \Vert _{L^{p}(\Omega_{i})} \\ &\leq \vert \Omega_{i} \vert ^{-1+\frac{1}{q'}} \Vert u \Vert _{L^{q}(\Omega_{i})} \vert \Omega_{i} \vert ^{\frac{1}{p}} \\ &= \vert \Omega_{i} \vert ^{\frac{1}{p}-\frac{1}{q}} \Vert u \Vert _{L^{q}(\Omega_{i})}. \end{aligned}$$

We describe the following proof separately for the case of $p=\infty$ and $p<\infty$.

When $p=\infty$, we have

$$\begin{aligned} \Vert u \Vert _{L^{\infty}(\Omega)}&=\max_{1\leq i\leq n} \Vert u \Vert _{L^{\infty}(\Omega_{i})} \\ &\leq\max_{1\leq i\leq n} \bigl( \Vert u_{\Omega_{i}} \Vert _{L^{\infty}(\Omega_{i})}+ \Vert u-u_{\Omega_{i}} \Vert _{L^{\infty}(\Omega_{i})} \bigr). \end{aligned}$$

From (5) and (7), it follows that

$$\begin{aligned} & \Vert u \Vert _{L^{\infty}(\Omega)} \\ &\quad \leq\max_{1\leq i\leq n} \bigl( \vert \Omega_{i} \vert ^{-\frac{1}{q}} \Vert u \Vert _{L^{q}(\Omega_{i})}+D_{\infty }(\Omega_{i}) \Vert \nabla u \Vert _{L^{q}(\Omega_{i})} \bigr) \\ &\quad \leq\max \Bigl\{ \max_{1\leq i\leq n} \vert \Omega_{i} \vert ^{-\frac{1}{q}}, \max_{1\leq i\leq n}D_{\infty}(\Omega _{i}) \Bigr\} \max_{1\leq i\leq n} \bigl( \Vert u \Vert _{L^{q}(\Omega_{i})}+ \Vert \nabla u \Vert _{L^{q}(\Omega _{i})} \bigr). \end{aligned}$$

This implies that Theorem 2.1 holds for the case of $p=\infty$ and $q=\infty$.

For $q<\infty$, we have

$$\begin{aligned} & \Vert u \Vert _{L^{\infty}(\Omega)} \\ &\quad \leq\max \Bigl\{ \max_{1\leq i\leq n} \vert \Omega_{i} \vert ^{-\frac{1}{q}}, \max_{1\leq i\leq n}D_{\infty}(\Omega _{i}) \Bigr\} \biggl(\sum_{1\leq i\leq n} \bigl( \Vert u \Vert _{L^{q}(\Omega_{i})}+ \Vert \nabla u \Vert _{L^{q}(\Omega _{i})} \bigr)^{q} \biggr)^{\frac{1}{q}} \\ &\quad \leq2^{1-\frac{1}{q}}\max \Bigl\{ \max_{1\leq i\leq n} \vert \Omega_{i} \vert ^{-\frac{1}{q}}, \max_{1\leq i\leq n}D_{\infty }( \Omega_{i}) \Bigr\} \Vert u \Vert _{W^{1,q}(\Omega)}, \end{aligned}$$

where the last inequality follows from $(s+t)^{q}\leq2^{q-1}(s^{q}+t^{q})$ for $s,t\geq0$.

When $p<\infty$, we have

$$\begin{aligned} \Vert u \Vert _{L^{p}(\Omega)}&= \biggl(\sum _{1\leq i\leq n} \int_{\Omega_{i}} \bigl\vert u(y) \bigr\vert ^{p}\,dy \biggr)^{\frac {1}{p}} \\ &= \biggl(\sum_{1\leq i\leq n} \Vert u \Vert _{L^{p}(\Omega _{i})}^{p} \biggr)^{\frac{1}{p}} \\ &\leq \biggl(\sum_{1\leq i\leq n} \bigl( \Vert u_{\Omega _{i}} \Vert _{L^{p}(\Omega_{i})}+ \Vert u-u_{\Omega_{i}} \Vert _{L^{p}(\Omega_{i})} \bigr)^{p} \biggr)^{\frac{1}{p}}. \end{aligned}$$

From (5) and (7), it follows that

$$\begin{aligned} \Vert u \Vert _{L^{p}(\Omega)}&\leq \biggl(\sum _{1\leq i\leq n} \bigl( \vert \Omega_{i} \vert ^{\frac{1}{p}-\frac {1}{q}} \Vert u \Vert _{L^{q}(\Omega_{i})}+D_{p}(\Omega _{i}) \Vert \nabla u \Vert _{L^{q}(\Omega_{i})} \bigr)^{p} \biggr)^{\frac{1}{p}} \\ &\leq \biggl(\sum_{1\leq i\leq n} \bigl( \vert \Omega_{i} \vert ^{\frac{1}{p}-\frac{1}{q}} \Vert u \Vert _{L^{q}(\Omega_{i})}+D_{p}(\Omega_{i}) \Vert \nabla u \Vert _{L^{q}(\Omega_{i})} \bigr)^{q} \biggr)^{\frac{1}{q}} \\ &\leq2^{1-\frac{1}{q}} \biggl(\sum_{1\leq i\leq n} \bigl( \vert \Omega_{i} \vert ^{\frac{q}{p}-1} \Vert u \Vert _{L^{q}(\Omega_{i})}^{q}+D_{p}(\Omega_{i})^{q} \Vert \nabla u \Vert _{L^{q}(\Omega_{i})}^{q} \bigr) \biggr)^{\frac{1}{q}}. \end{aligned}$$

Therefore, we obtain

$$\begin{aligned} \Vert u \Vert _{L^{p}(\Omega)}\leq2^{1-\frac{1}{q}}\max \Bigl\{ \max_{1\leq i\leq n} \vert \Omega_{i} \vert ^{\frac {1}{p}-\frac{1}{q}}, \max_{1\leq i\leq n}D_{i}( \Omega_{i}) \Bigr\} \Vert u \Vert _{W^{1,q}(\Omega)}. \end{aligned}$$

 □

## Estimation of $D_{p}(\Omega_{i})$

Let Γ be the gamma function, that is, $\Gamma(x)=\int _{0}^{\infty}t^{x-1}e^{-t}\,dt$ for $x>0$. For $f\in L^{r}(\mathbb{R}^{N})$ and $g\in L^{s}(\mathbb{R}^{N})$ ($1\leq r,s\leq\infty$), let $f*g: \mathbb{R}^{N}\to\mathbb{R}$ be the convolution of *f* and *g* defined by

$$\begin{aligned} (f*g) (x):= \int_{\mathbb{R}^{N}}f(x-y)g(y)\,dy \biggl(= \int_{\mathbb {R}^{N}}f(x)g(x-y)\,dy \biggr). \end{aligned}$$

In the following three lemmas, we recall some known results required to obtain explicit values of $D_{p}(\Omega_{i})$ in (5) for bounded convex domains $\Omega_{i}$.

### Lemma 3.1

(see, e.g., [30, 31])

*Let* $\Omega\subset\mathbb{R}^{N}$ ($N\in\mathbb{N}$) *be a bounded convex domain*. *For* $u\in W^{1,1}(\Omega)$ *and any point* $x\in\Omega $, *we have*

$$\begin{aligned} \bigl\vert u(x)-u_{\Omega}(x) \bigr\vert \leq\frac{d_{\Omega }^{N}}{N \vert \Omega \vert } \int_{\Omega} \vert x-y \vert ^{1-N} \bigl\vert \nabla u(y) \bigr\vert \,dy. \end{aligned}$$

A proof of Lemma 3.1 is provided in Appendix 2 because Lemma 3.1 plays an especially important role in obtaining the explicit values of $D_{p}(\Omega_{i})$.

### Lemma 3.2

(Hardy-Littlewood-Sobolev’s inequality [32])

*For* $\lambda>0$, *we put* $h_{\lambda}(x):= \vert x \vert ^{-\lambda}$. *If* $0<\lambda<N$,

8 $$\begin{aligned} \Vert h_{\lambda}*g \Vert _{L^{\frac{2N}{\lambda }}(\mathbb{R}^{N})}\leq C_{\lambda, N} \Vert g \Vert _{L^{\frac{2N}{2N-\lambda}}(\mathbb{R}^{N})}\quad \textit{for all } g\in L^{\frac{2N}{2N-\lambda}}\bigl(\mathbb{R}^{N}\bigr) \end{aligned}$$

*holds valid for*

9 $$\begin{aligned} C_{\lambda, N}=\pi^{\frac{\lambda}{2}}\frac{\Gamma(\frac {N}{2}-\frac{\lambda}{2})}{\Gamma(N-\frac{\lambda}{2})} \biggl( \frac{\Gamma(\frac{N}{2})}{\Gamma(N)} \biggr)^{-1+\frac{\lambda}{N}}, \end{aligned}$$

*where this is the best constant in* (8).

*Moreover*, *if* $N<2\lambda<2N$,

10 $$\begin{aligned} \Vert h_{\lambda}*g \Vert _{L^{\frac{2N}{2\lambda -N}}(\mathbb{R}^{N})}\leq\tilde{C}_{\lambda, N} \Vert g \Vert _{L^{2}(\mathbb{R}^{N})}\quad \textit{for all } g\in L^{2}\bigl(\mathbb{R}^{N}\bigr) \end{aligned}$$

*holds valid for*

11 $$\begin{aligned} \tilde{C}_{\lambda, N}=\pi^{\frac{\lambda}{2}}\frac{\Gamma(\frac {N}{2}-\frac{\lambda}{2})}{\Gamma(\frac{\lambda}{2})} \sqrt{\frac {\Gamma(\lambda-\frac{N}{2})}{\Gamma(\frac{3N}{2}-\lambda)}} \biggl(\frac{\Gamma(\frac{N}{2})}{\Gamma(N)} \biggr)^{-1+\frac{\lambda}{N}}, \end{aligned}$$

*where this is the best constant in* (10).

### Lemma 3.3

(Young’s inequality [33])

*Suppose that* $1\leq t,r,s\leq\infty$ *and* $1/t=1/r+1/s-1\geq0$. *For* $f\in L^{r}(\mathbb{R}^{N})$ *and* $g\in L^{s}(\mathbb{R}^{N})$, *we have*

12 $$\begin{aligned} \Vert f*g \Vert _{L^{t}(\mathbb{R}^{N})}\leq (A_{r}A_{s}A_{t'})^{N} \Vert f \Vert _{L^{r}(\mathbb {R}^{N})} \Vert g \Vert _{L^{s}(\mathbb{R}^{N})} \end{aligned}$$

*with*

$$\begin{aligned} A_{m}= \textstyle\begin{cases} \sqrt{m^{\frac{2}{m}-1}(m-1)^{1-\frac{1}{m}}}&(1< m< \infty),\\ 1&(m=1, \infty). \end{cases}\displaystyle \end{aligned}$$

*The constant* $(A_{r}A_{s}A_{t'})^{N}$ *is the best constant in* (12).

The following Theorems 3.1, 3.2, 3.3, and 3.4 provide estimations of $D_{p}(\Omega)$ for a bounded convex domain Ω, where *p*, *q*, and *N* are imposed on the assumptions listed in Table 1.

### Theorem 3.1

*Let* $\Omega\subset\mathbb{R}^{N}$ ($N\in\mathbb{N}$) *be a bounded convex domain*. *Assume that* $p\in\mathbb{R}$ *satisfies* $2< p\leq 2N/(N-1)$ *if* $N\geq2$ *and* $2< p<\infty$ *if* $N=1$. *For* $q\in\mathbb{R}$ *such that* $q\geq p/(p-1)$, *we have*

$$\begin{aligned} \Vert u-u_{\Omega} \Vert _{L^{p}(\Omega)}\leq D_{p}(\Omega ) \Vert \nabla u \Vert _{L^{q}(\Omega)} \quad \textit{for all } u\in W^{1,q}(\Omega) \end{aligned}$$

*with*

$$\begin{aligned} D_{p}(\Omega)=\frac{d_{\Omega}^{1+\frac{2N}{p}}\pi^{\frac {N}{p}}}{N \vert \Omega \vert ^{\frac{1}{p}+\frac {1}{q}}}\frac{\Gamma(\frac{p-2}{2p}N)}{\Gamma(\frac {p-1}{p}N)} \biggl( \frac{\Gamma(N)}{\Gamma(\frac{N}{2})} \biggr)^{\frac{p-2}{p}}. \end{aligned}$$

### Proof

Let $u\in W^{1,q}(\Omega)$. Since $p\leq2N/(N-1)$ and $1-N+(2N/p)\geq 0$, it follows that $\vert x-z \vert ^{1-N+\frac {2N}{p}}\leq d_{\Omega}^{1-N+\frac{2N}{p}}$ for $x, z\in\Omega$. Lemma 3.1 implies that, for a fixed $x\in\Omega$,

$$\begin{aligned} \bigl\vert u(x)-u_{\Omega}(x) \bigr\vert &\leq \frac{d_{\Omega }^{N}}{N \vert \Omega \vert } \int_{\Omega} \vert x-z \vert ^{1-N+\frac{2N}{p}} \vert x-z \vert ^{-\frac{2N}{p}} \bigl\vert \nabla u(z) \bigr\vert \,dz \\ &\leq\frac{d_{\Omega}^{1+\frac{2N}{p}}}{N \vert \Omega \vert } \int_{\Omega} \vert x-z \vert ^{-\frac {2N}{p}} \bigl\vert \nabla u(z) \bigr\vert \,dz \\ &\leq\frac{d_{\Omega}^{1+\frac{2N}{p}}}{N \vert \Omega \vert } \int_{\mathbb{R}^{N}} \vert x-z \vert ^{-\frac {2N}{p}} \bigl(E_{\Omega,\mathbb{R}^{N}} \vert \nabla u \vert \bigr) (z)\,dz. \end{aligned} $$

Therefore,

$$\begin{aligned} \Vert u-u_{\Omega} \Vert _{L^{p}(\Omega)}&\leq\frac {d_{\Omega}^{1+\frac{2N}{p}}}{N \vert \Omega \vert } \biggl( \int_{\Omega} \biggl( \int_{\mathbb{R}^{N}} \vert x-z \vert ^{-\frac{2N}{p}} \bigl(E_{\Omega,\mathbb{R}^{N}} \vert \nabla u \vert \bigr) (z)\,dz \biggr)^{p}\,dx \biggr)^{\frac{1}{p}} \\ &\leq\frac{d_{\Omega}^{1+\frac{2N}{p}}}{N \vert \Omega \vert } \biggl( \int_{\mathbb{R}^{N}} \biggl( \int_{\mathbb{R}^{N}} \vert x-z \vert ^{-\frac{2N}{p}} \bigl(E_{\Omega,\mathbb {R}^{N}} \vert \nabla u \vert \bigr) (z)\,dz \biggr)^{p}\,dx \biggr)^{\frac{1}{p}}. \end{aligned}$$

Since $q\geq p/(p-1)$ and Ω is bounded, we have $\vert \nabla u \vert \in L^{p/(p-1)}(\Omega)$. Therefore, Lemma 3.2 ensures

$$\begin{aligned} \Vert u-u_{\Omega} \Vert _{L^{p}(\Omega)}&\leq\frac {d_{\Omega}^{1+\frac{2N}{p}}}{N \vert \Omega \vert }C_{\frac{2N}{p}, N} \bigl\Vert E_{\Omega,\mathbb{R}^{N}} \vert \nabla u \vert \bigr\Vert _{L^{\frac{p}{p-1}}(\mathbb{R}^{N})} \\ &=\frac{d_{\Omega}^{1+\frac{2N}{p}}}{N \vert \Omega \vert }C_{\frac{2N}{p}, N} \Vert \nabla u \Vert _{L^{\frac {p}{p-1}}(\Omega)}, \end{aligned}$$

where $C_{\frac{2N}{p}, N}$ is defined in (9) with $\lambda=2N/p$. Since $q\geq p/(p-1)$, Hölder’s inequality moreover implies

$$\begin{aligned} \Vert u-u_{\Omega} \Vert _{L^{p}(\Omega)}&\leq\frac {d_{\Omega}^{1+\frac{2N}{p}}}{N \vert \Omega \vert ^{\frac{1}{p}+\frac{1}{q}}}C_{\frac{2N}{p}, N} \Vert \nabla u \Vert _{L^{q}(\Omega)}. \end{aligned}$$

 □

### Theorem 3.2

*Let* $\Omega\subset\mathbb{R}^{N}$ ($N\geq2$) *be a bounded convex domain*. *Assume that* $2< p\leq2N/(N-2)$ *if* $N\geq3$ *and* $2< p<\infty$ *if* $N=2$. *For all* $u\in W^{1,2}(\Omega)$, *we have*

$$\begin{aligned} \Vert u-u_{\Omega} \Vert _{L^{p}(\Omega)}\leq D_{p}(\Omega ) \Vert \nabla u \Vert _{L^{2}(\Omega)} \end{aligned}$$

*with*

$$\begin{aligned} D_{p}(\Omega)=\frac{d_{\Omega}^{1+\frac{p+2}{2p}N}\pi^{\frac {p+2}{4p}N}}{N \vert \Omega \vert }\frac{\Gamma(\frac {p-2}{4p}N)}{\Gamma(\frac{p+2}{4p}N)}\sqrt{ \frac{\Gamma(\frac {N}{p})}{\Gamma(\frac{p-1}{p}N)}} \biggl(\frac{\Gamma(N)}{\Gamma (\frac{N}{2})} \biggr)^{\frac{p-2}{2p}}. \end{aligned}$$

### Proof

Let $u\in W^{1,2}(\Omega)$. Since $p\leq2N/(N-2)$, it follows that $\vert x-z \vert ^{1-N+(p+2)N/(2p)}\leq d_{\Omega }^{1-N+(p+2)N/(2p)}$ for $x, z\in\Omega$. Lemma 3.1 leads to

$$\begin{aligned} \bigl\vert u(x)-u_{\Omega}(x) \bigr\vert &\leq \frac{d_{\Omega }^{N}}{N \vert \Omega \vert } \int_{\Omega} \vert x-z \vert ^{1-N+\frac{p+2}{2p}N} \vert x-z \vert ^{-\frac{p+2}{2p}N} \bigl\vert \nabla u(z) \bigr\vert \,dz \\ &\leq\frac{d_{\Omega}^{1+\frac{p+2}{2p}N}}{N \vert \Omega \vert } \int_{\Omega} \vert x-z \vert ^{-\frac {p+2}{2p}N} \bigl\vert \nabla u(z) \bigr\vert \,dz \\ &\leq\frac{d_{\Omega}^{1+\frac{p+2}{2p}N}}{N \vert \Omega \vert } \int_{\mathbb{R}^{N}} \vert x-z \vert ^{-\frac {p+2}{2p}N} \bigl(E_{\Omega,\mathbb{R}^{N}} \vert \nabla u \vert \bigr) (z)\,dz. \end{aligned} $$

Therefore,

$$\begin{aligned} \Vert u-u_{\Omega} \Vert _{L^{p}(\Omega)}&\leq\frac {d_{\Omega}^{1+\frac{p+2}{2p}N}}{N \vert \Omega \vert } \biggl( \int_{\Omega} \biggl( \int_{\mathbb{R}^{N}} \vert x-z \vert ^{-\frac{p+2}{2p}N} \bigl(E_{\Omega,\mathbb {R}^{N}} \vert \nabla u \vert \bigr) (z)\,dz \biggr)^{p}\,dx \biggr)^{\frac{1}{p}} \\ &\leq\frac{d_{\Omega}^{1+\frac{p+2}{2p}N}}{N \vert \Omega \vert } \biggl( \int_{\mathbb{R}^{N}} \biggl( \int_{\mathbb {R}^{N}} \vert x-z \vert ^{-\frac{p+2}{2p}N} \bigl(E_{\Omega ,\mathbb{R}^{N}} \vert \nabla u \vert \bigr) (z)\,dz \biggr)^{p}\,dx \biggr)^{\frac{1}{p}}. \end{aligned}$$

From (10), it follows that

$$\begin{aligned} \Vert u-u_{\Omega} \Vert _{L^{p}(\Omega)}&\leq\frac {d_{\Omega}^{1+\frac{p+2}{2p}N}}{N \vert \Omega \vert }\tilde{C}_{\frac{p+2}{2p}N, N} \bigl\Vert E_{\Omega,\mathbb {R}^{N}} \vert \nabla u \vert \bigr\Vert _{L^{2}(\mathbb {R}^{N})} \\ &=\frac{d_{\Omega}^{1+\frac{p+2}{2p}N}}{N \vert \Omega \vert }\tilde{C}_{\frac{p+2}{2p}N, N} \Vert \nabla u \Vert _{L^{2}(\Omega)}, \end{aligned}$$

where $\tilde{C}_{\frac{p+2}{2p}N, N}$ is defined in (11) with $\lambda=(p+2)N/(2p)$. □

### Theorem 3.3

*Let* $\Omega\subset\mathbb{R}^{N}$ ($N\in\mathbb{N}$) *be a bounded convex domain*. *Suppose that* $1\leq q\leq p< qN/(N-q)$ *if* $N>q$, *and* $1\leq q\leq p<\infty$ *if* $N=q$. *Then we have*

13 $$\begin{aligned} \Vert u-u_{\Omega} \Vert _{L^{p}(\Omega)}\leq D_{p}(\Omega ) \Vert \nabla u \Vert _{L^{q}(\Omega)}\quad \textit{for all } u\in W^{1,q}(\Omega) \end{aligned}$$

*with*

$$\begin{aligned} D_{p}(\Omega)=\frac{d_{\Omega}^{N}}{N \vert \Omega \vert }(A_{r}A_{q}A_{p'})^{N} \bigl\Vert \vert x \vert ^{1-N} \bigr\Vert _{L^{r}(V)}, \end{aligned}$$

*where* $\Omega_{x}:=\{x-y\mid y\in\Omega\}$ *for* $x\in\Omega$, $V:=\bigcup_{x\in\Omega}\Omega_{x}$, *and* $r=qp/((q-1)p+q)$.

### Proof

First, we prove $I:= \Vert \vert x \vert ^{1-N} \Vert _{L^{r}(V)}^{r}<\infty$. Let $\rho=2d_{\Omega}$ so that $V\subset B(0,\rho)$. We have

$$\begin{aligned} \frac{pq(1-N)}{(q-1)p+q}+N-1&=\frac{pq(1-N)+Np(q-1)+Nq}{(q-1)p+q}-1 \\ &=\frac{Nq-(N-q)p}{(q-1)p+q}-1>-1. \end{aligned}$$

Therefore,

$$\begin{aligned} I&= \int_{V} \vert x \vert ^{\frac{pq(1-N)}{(q-1)p+q}}\,dx \leq \int_{B(0,\rho)} \vert x \vert ^{\frac{pq(1-N)}{(q-1)p+q}}\,dx =J \int_{0}^{\rho}\rho^{\frac{pq(1-N)}{(q-1)p+q}+N-1}\,d\rho < \infty, \end{aligned}$$

where *J* is defined by

$$J= \textstyle\begin{cases} 2&(N=1),\\ 2\pi&(N=2),\\ 2\pi\int_{[0,\pi]^{N-2}}\prod_{i=1}^{N-2}(\sin\theta_{i})^{N-i-1} \,d\theta_{1}\cdots \,d\theta_{N-2}&(N\geq3). \end{cases} $$

Next, we show (13). For $x\in\Omega$, it follows from Lemma 3.1 that

$$\begin{aligned} \bigl\vert u(x)-u_{\Omega}(x) \bigr\vert &\leq\frac{d_{\Omega }^{N}}{N \vert \Omega \vert } \int_{\Omega} \vert x-y \vert ^{1-N} \bigl\vert \nabla u(y) \bigr\vert \,dy \\ &=\frac{d_{\Omega}^{N}}{N \vert \Omega \vert } \int_{\Omega _{x}} \vert y \vert ^{1-N} \bigl\vert \nabla u(x-y) \bigr\vert \,dy \\ &\leq\frac{d_{\Omega}^{N}}{N \vert \Omega \vert } \int _{V} \vert y \vert ^{1-N} \bigl(E_{\Omega,V} \vert \nabla u \vert \bigr) (x-y)\,dy. \end{aligned}$$

Since $E_{V,\mathbb{R}^{N}}E_{\Omega,V}=E_{\Omega,\mathbb{R}^{N}}$,

14 $$\begin{aligned} \bigl\vert u(x)-u_{\Omega}(x) \bigr\vert &\leq \frac{d_{\Omega }^{N}}{N \vert \Omega \vert } \int_{\mathbb{R}^{N}} (E_{V,\mathbb{R}^{N}}\psi ) (y) \bigl(E_{\Omega,\mathbb {R}^{N}} \vert \nabla u \vert \bigr) (x-y)\,dy, \end{aligned}$$

where $\psi(y)= \vert y \vert ^{1-N}$ for $y\in V$. We denote $f(x)= (E_{V,\mathbb{R}^{N}}\psi )(x)$ and $g(x)= (E_{\Omega,\mathbb{R}^{N}} \vert \nabla u \vert )(x)$. Lemma 3.3 and (14) give

$$\begin{aligned} \Vert u-u_{\Omega} \Vert _{L^{p}(\Omega)}&\leq\frac {d_{\Omega}^{N}}{N \vert \Omega \vert } \Vert f*g \Vert _{L^{p}(\Omega)} \\ &\leq\frac{d_{\Omega}^{N}}{N \vert \Omega \vert } \Vert f*g \Vert _{L^{p}(\mathbb{R}^{N})} \\ &\leq\frac{d_{\Omega}^{N}}{N \vert \Omega \vert }(A_{r}A_{q}A_{p'})^{N} \Vert f \Vert _{L^{r}(\mathbb {R}^{N})} \Vert g \Vert _{L^{q}(\mathbb{R}^{N})} \\ &=\frac{d_{\Omega}^{N}}{N \vert \Omega \vert }(A_{r}A_{q}A_{p'})^{N} I^{\frac{1}{r}} \Vert \nabla u \Vert _{L^{q}(\Omega)}. \end{aligned}$$

 □

### Theorem 3.4

*Let* $\Omega\subset\mathbb{R}^{N}$ ($N\in\mathbb{N}$) *be a bounded convex domain*, *and let* $q>N$. *Then we have*

15 $$\begin{aligned} \Vert u-u_{\Omega} \Vert _{L^{\infty}(\Omega)}\leq D_{\infty}(\Omega) \Vert \nabla u \Vert _{L^{q}(\Omega )}\quad \textit{for all } u\in W^{1,q}(\Omega) \end{aligned}$$

*with*

$$\begin{aligned} D_{\infty}(\Omega)=\frac{d_{\Omega}^{N}}{N \vert \Omega \vert } \bigl\Vert \vert x \vert ^{1-N} \bigr\Vert _{L^{q'}(V)}, \end{aligned}$$

*where* *V* *is defined in Theorem* 3.3.

### Proof

First, we show $I:= \Vert \vert x \vert ^{1-N} \Vert _{L^{q'}(V)}^{q'}<\infty$. Let $\rho=2d_{\Omega}$ so that $V\subset B(0,\rho)$. We have

$$\begin{aligned} q'(1-N)+N-1=\frac{q(1-N)+N(q-1)}{q-1}-1=\frac{q-N}{q-1}-1>-1. \end{aligned}$$

Therefore,

$$\begin{aligned} I= \int_{V} \vert x \vert ^{q'(1-N)}\,dx\leq \int_{B(0,\rho )} \vert x \vert ^{q'(1-N)}\,dx =J \int_{0}^{\rho}\rho^{q'(1-N)+N-1}\,d\rho < \infty, \end{aligned}$$

where *J* is defined in the proof of Theorem 3.3.

Next, we prove (15). Let $r=\frac{q}{q-1}(\geq1)$, $f(x)= (E_{V,\mathbb{R}^{N}}\psi )(x)$, and $g(x)= (E_{\Omega,\mathbb{R}^{N}} \vert \nabla u \vert )(x)$, where *ψ* is denoted in the proof of Theorem 3.3. From Lemma 3.3 and (14), for $u\in W^{1,q}(\Omega)$, it follows that

$$\begin{aligned} \Vert u-u_{\Omega} \Vert _{L^{\infty}(\Omega)}&\leq\frac {d_{\Omega}^{N}}{N \vert \Omega \vert } \Vert f*g \Vert _{L^{\infty}(\Omega)} \leq\frac{d_{\Omega}^{N}}{N \vert \Omega \vert } \Vert f*g \Vert _{L^{\infty}(\mathbb{R}^{N})} \\ &\leq\frac{d_{\Omega}^{N}}{N \vert \Omega \vert } \Vert f \Vert _{L^{q'}(\mathbb{R}^{N})} \Vert g \Vert _{L^{q}(\mathbb{R}^{N})} =\frac{d_{\Omega}^{N}}{N \vert \Omega \vert }I^{\frac {1}{q'}} \Vert \nabla u \Vert _{L^{q}(\Omega)}. \end{aligned}$$

 □

## Explicit values of $C_{p}(\Omega)$ for certain domains

In this section, we present numerical examples where explicit values of $C_{p}(\Omega)$ on a square and a triangle domain are computed using Theorems 2.1, 3.1, 3.2, 3.3, and 3.4. All computations were performed on a computer with Intel Xeon E5-2687W @ 3.10 GHz, 512 GB RAM, CentOS 7, and MATLAB 2017a. All rounding errors were strictly estimated using the interval toolbox INTLAB version 10.1 [34]. Therefore, all values in the following tables are mathematically guaranteed to be upper bounds of the corresponding $C_{p}(\Omega)$’s.

First, we select domains $\Omega_{i}$ ($1\leq i\leq n$) satisfying (2) and (3). For all domains $\Omega_{i}$ ($1\leq i\leq n$), we then compute the values of $D_{p}(\Omega_{i})$ using Theorems 3.1, 3.2, 3.3, and 3.4. Next, explicit values of $C_{p}(\Omega)$ are computed through Theorem 2.1.

### Estimation on a square domain

**Figure 1:**
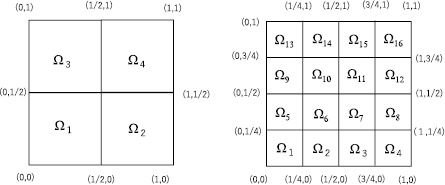
$\pmb{\Omega_{i}}$ **for the cases in which** $\pmb{n=4}$ **(the left-hand side) and** $\pmb{n=16}$ **(the right-hand side).**

For the first example, we select the case in which $\Omega=(0,1)^{2}$. For $n=1,4,16, 64, \ldots$ , we define each $\Omega_{i}$ ($1\leq i\leq n$) as a square with side length $1/\sqrt{n}$; see Figure 1 for the cases in which $n=4$ and $n=16$. For this division of Ω, Theorem 2.1 states that

$$\begin{aligned} C_{p}(\Omega)=2^{1-\frac{1}{q}}\max \Bigl(n^{- (\frac {1}{p}-\frac{1}{q} )}, \max _{1\leq i\leq n}D_{p}(\Omega_{i}) \Bigr). \end{aligned}$$

**Figure 2:**
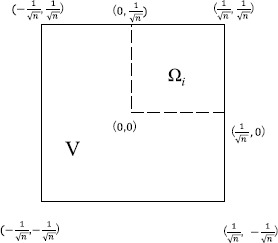
**The domain** ***V*** **in Theorems** **3.3** **and** **3.4** **.**

In this case, *V* (in Theorems 3.3 and 3.4) becomes a square with side length $2/\sqrt{n}$ (see Figure 2). Note that $\Vert \vert x \vert ^{1-N} \Vert _{L^{r}(V)}=\int_{V} \vert x \vert ^{\beta}\,dx$, where $\beta =qp(1-N)/((q-1)p+q)$ if $p<\infty$ and $\beta=q'(1-N)$ if $p=\infty$.

**Figure 3:**
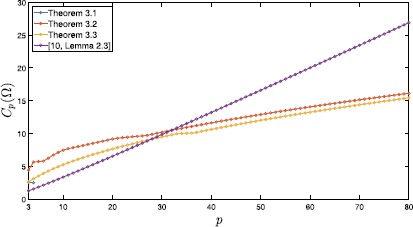
**Computed values of** $\pmb{C_{p}(\Omega)}$ **for** $\pmb{\Omega =(0,1)^{2}}$ **and** $\pmb{3\leq p\leq80}$ **.**

Table 2 compares upper bounds for $C_{p}(\Omega)$ computed by Theorems 3.1, 3.2, 3.3, [10, Lemma 2.3], and [19, Corollary D.1] with $q=2$; the numbers of division *n* are shown in the corresponding parentheses. Moreover, these values are plotted in Figure 3, except for the values derived from [19, Corollary D.1].

**Table 2:** **Computed values of** $\pmb{C_{p}(\Omega)}$ **for** $\pmb{\Omega=(0,1)^{2}}$ **and** $\pmb{q=2}$ **. The numbers of division** ***n*** **are shown in the corresponding parentheses. Theorem** **3.1** **cannot be used for** $\pmb{p>4}$ **when** $\pmb{N=2}$

***p***	**Theorem ** **3.1**	**Theorem ** **3.2**	**Theorem ** **3.3**	**[** 10 **, Lemma 2.3]**	**[** 19 **, Corollary D.1]**
3	2.553767 (16)	4.423506 (256)	2.647076 (16)	1.272533	1.291703 × 10^4^
4	2.506629 (4)	5.656855 (256)	3.098954 (16)	1.553774	1.809271 × 10^4^
5	-	5.721912 (64)	3.527578 (16)	1.841950	2.275458 × 10^4^
6	-	5.802230 (64)	3.922709 (16)	2.135792	2.701890 × 10^4^
7	-	6.245674 (64)	4.288114 (16)	2.434362	3.096661 × 10^4^
8	-	6.727172 (64)	4.628497 (16)	2.736941	3.465528 × 10^4^
9	-	7.127190 (64)	4.947849 (16)	3.042967	3.812726 × 10^4^
10	-	7.464264 (64)	5.249352 (16)	3.351991	4.141471 × 10^4^
20	-	9.162396 (16)	7.659208 (16)	6.549949	6.789009 × 10^4^
30	-	10.202188 (64)	9.485455 (16)	9.856546	8.800592 × 10^4^
40	-	11.632217 (64)	10.640059 (64)	13.218367	1.048141 × 10^5^
50	-	12.907885 (64)	12.020066 (64)	16.613831	1.195208 × 10^5^
60	-	14.069728 (64)	13.258962 (64)	20.031993	1.327453 × 10^5^
70	-	15.143396 (64)	14.392550 (64)	23.466517	1.448540 × 10^5^
80	-	16.146231 (64)	15.443710 (64)	26.913400	1.560849 × 10^5^

Theorems 3.1, 3.2, 3.3, and [10, Lemma 2.3] provide sharper estimates of $C_{p}(\Omega)$ than [19, Corollary D.1] for all *p*’s. The estimates derived by Theorem 3.2 and Theorem 3.3 for $32\leq p\leq80$ are sharper than the estimates obtained by [10, Lemma 2.3].

We also show the values of $C_{\infty}(\Omega)$ computed by Theorem 3.4 for $3\leq q\leq10$ in Table 3.

**Table 3:** **Computed values of** $\pmb{C_{\infty}(\Omega)}$ **for a square domain** **Ω** **and** $\pmb{3\leq q\leq10}$ **. The numbers of division** ***n*** **are shown in the corresponding parentheses**

***q***	**Theorem ** **3.4**
3	5.611920 (16)
4	4.756829 (64)
5	4.000001 (64)
6	3.563595 (64)
7	3.281342 (64)
8	3.084422 (64)
9	2.939469 (64)
10	2.828428 (64)

### Estimation on a triangle domain

**Figure 4:**
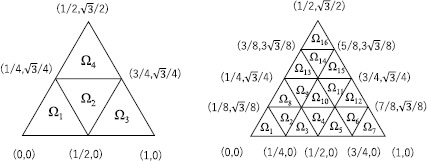
$\pmb{\Omega_{i}}$ **when** $\pmb{n=4}$ **(the left-hand side) and** $\pmb{n=16}$ **(the right-hand side).**

For the second example, we select the case in which Ω is a regular triangle with the vertices $(0,0)$, $(1,0)$, and $(1/2,\sqrt {3}/2)$. For $n=1,4,16,64,\ldots$ , we define each $\Omega_{i}$ ($1\leq i\leq n$) as a regular triangle with side length $1/\sqrt{n}$; see Figure 4 for the case in which $n=4$ and $n=16$. For this division of Ω, Theorem 2.1 states that

$$C_{p}(\Omega)=2^{1-\frac{1}{q}}\max \biggl( \biggl(\frac{4n}{\sqrt {3}} \biggr)^{- (\frac{1}{p}-\frac{1}{q} )}, \max_{1\leq i\leq n} D_{p}( \Omega_{i}) \biggr). $$

**Figure 5:**
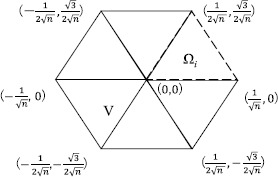
**The domain** ***V*** **in Theorems** **3.3** **and** **3.4** **.**

In this case, *V* is the regular hexagon displayed in Figure 5.

**Figure 6:**
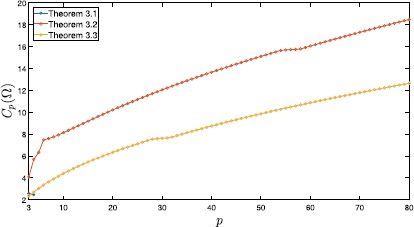
**Computed values of** $\pmb{C_{p}(\Omega)}$ **for a regular triangle domain** **Ω** **and** $\pmb{3\leq p\leq80}$ **.**

Table 4 compares upper bounds of $C_{p}(\Omega)$ computed by Theorems 3.1, 3.2, 3.3, and [19, Corollary D.1] with $q=2$; the numbers of division *n* are shown in the corresponding parentheses. Moreover, these values are plotted in Figure 6. The estimate computed by Theorem 3.1 is sharpest when $p=4$. However, for the other *p* satisfying $3\leq p\leq80$, Theorem 3.3 provides the sharpest estimates.

**Table 4:** **Computed values of** $\pmb{C_{p}(\Omega)}$ **for a regular triangle domain** **Ω** **and** $\pmb{q=2}$ **. The numbers of division** ***n*** **are shown in the corresponding parentheses. Theorem** **3.1** **cannot be used for** $\pmb{p>4}$ **when** $\pmb{N=2}$

***p***	**Theorem ** **3.1**	**Theorem ** **3.2**	**Theorem ** **3.3**	**[** 19 **, Corollary D.1]**
3	2.580982 (16)	4.097053 (256)	2.366856 (4)	2.538335 × 10^4^
4	2.465500 (4)	5.700515 (64)	2.709475 (4)	3.553398 × 10^4^
5	-	6.330220 (64)	3.042818 (4)	4.464990 × 10^4^
6	-	7.477243 (64)	3.353176 (4)	5.297547 × 10^4^
7	-	7.601403 (16)	3.641844 (4)	6.067602 × 10^4^
8	-	7.750471 (16)	3.911816 (4)	6.786738 × 10^4^
9	-	7.933346 (16)	4.165864 (4)	7.463399 × 10^4^
10	-	8.133664 (16)	4.406282 (4)	8.103954 × 10^4^
20	-	10.219436 (16)	6.341217 (4)	1.326097 × 10^5^
30	-	12.055827 (16)	7.622031 (16)	1.717928 × 10^5^
40	-	13.666509 (16)	8.748299 (16)	2.045371 × 10^5^
50	-	15.112804 (16)	9.869218 (16)	2.331904 × 10^5^
60	-	16.059718 (64)	10.876336 (16)	2.589578 × 10^5^
70	-	17.313793 (64)	11.798394 (16)	2.825529 × 10^5^
80	-	18.483221 (64)	12.653794 (16)	3.044383 × 10^5^

We also show the values of $C_{\infty}(\Omega)$ computed by Theorem 3.4 for $3\leq q\leq10$ in Table 5.

**Table 5:** **Computed values of** $\pmb{C_{\infty}(\Omega)}$ **for a regular triangle domain** **Ω** **and** $\pmb{3\leq q\leq10}$ **. The numbers of division** ***n*** **are shown in the corresponding parentheses**

***q***	**Theorem ** **3.4**
3	4.797133 (4)
4	4.146459 (16)
5	3.583834 (16)
6	3.251833 (16)
7	3.033691 (16)
8	2.879743 (16)
9	2.765427 (16)
10	2.677251 (16)

#### Remark 4.1

**Figure 7:**
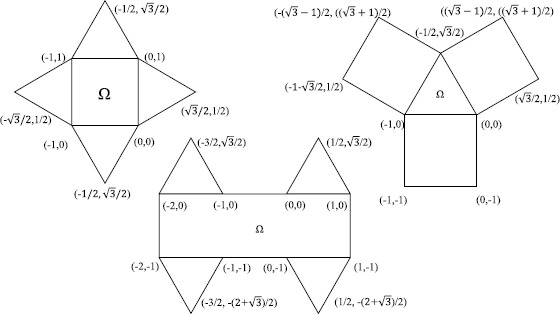
**Some examples of domains** **Ω** **that are composed of unit squares and triangles with side length** **1.**

The values of $C_{p}(\Omega)$ derived from Theorem 3.1 to 3.4 (provided in Tables 1 to 5) can be directly used for any domain that is composed of unit squares and triangles with side length 1 (see Figure 7 for some examples).

### Estimation on a cube domain

For the third example, we select the case in which $\Omega=(0,1)^{3}$. For $n=1,8,64,512,\ldots$ , we define each $\Omega_{i}$ ($1\leq i\leq n$) as a cube with side length $1/\sqrt[3]{n}$. For this division of Ω, Theorem 2.1 states that

$$C_{p}(\Omega)=2^{1-\frac{1}{q}}\max \Bigl(n^{- (\frac {1}{p}-\frac{1}{q} )}, \max _{1\leq i\leq n} D_{p}(\Omega _{i}) \Bigr). $$

In this case, *V* is also a cube with the side length $2/\sqrt[3]{n}$.

Table 6 compares upper bounds of $C_{p}(\Omega)$ computed by Theorems 3.1, 3.2, 3.3, and [19, Corollary D.1] with $q=2$; the numbers of division *n* are shown in the corresponding parentheses. The minimum value for each *p* is written in bold. We also show the values of $C_{\infty}(\Omega)$ computed by Theorem 3.4 for $4\leq q\leq10$ in Table 7.

**Table 6:** **Computed values of** $\pmb{C_{p}(\Omega)}$ **for a cube domain** **Ω** **and** $\pmb{q=2}$ **. The numbers of division** ***n*** **are shown in the corresponding parentheses. Theorem** **3.1** **for** $\pmb{p>3}$ **cannot be used when** $\pmb{N=3}$ **. Theorem** **3.2** **can be used for** $\pmb{p=6}$ **only when** $\pmb{N=3}$

***p***	**Theorem ** **3.1**	**Theorem ** **3.2**	**Theorem ** **3.3**	**[** 19 **, Corollary D.1]**
3	**4.000001** (512)	10.919242 (32,768)	5.947133 (4096)	3.115606 × 10^4^
4	-	16.340789 (4096)	**13.241245** (4096)	4.219101 × 10^4^
5	-	**18.436348** (4096)	29.676745 (4096)	5.239741 × 10^4^
6	-	**20.658471** (1)	−	-

**Table 7:** **Computed values of** $\pmb{C_{\infty}(\Omega)}$ **for a cube domain** **Ω** **and** $\pmb{4\leq q\leq10}$ **. The numbers of division** ***n*** **are shown in the corresponding parentheses**

***q***	**Theorem ** **3.4**
4	22.627417 (32,768)
5	13.928810 (32,768)
6	10.079369 (32,768)
7	8.000001 (32,768)
8	6.727172 (32,768)
9	5.878938 (32,768)
10	5.278032 (32,768)

## Conclusion

We proposed several theorems that provide explicit values of Sobolev type embedding constant $C_{p}(\Omega)$ satisfying (1) for a domain Ω that can be divided into a finite number of bounded convex domains. These theorems give sharper estimates of $C_{p}(\Omega)$ than the previous estimates derived by the method in [19]. This accuracy improvement leads to much applicability of the estimates of $C_{p}(\Omega)$ to verified numerical computations for PDEs.

## Appendix 1: Embedding constant $C_{p}(\Omega)$ on dividable domains

Theorem 6.1 provides an estimation of the embedding constant $C_{p}(\Omega)$ for a domain Ω that can be divided into domains $\Omega_{i}$ (such as convex domains and Lipschitz domains) satisfying (2) and (3).

### Theorem 6.1

*Let* $\Omega\subset\mathbb{R}^{N}$ ($N\in\mathbb{N}$) *be a domain that can be divided into a finite number of domains* $\Omega _{i}$ ($i=1,2,3,\ldots, n$) *satisfying* (2) *and* (3). *Assume that*, *for every* $\Omega_{i}$ ($i=1,2,3,\ldots, n$), *there exists a constant* $C_{p}(\Omega_{i})$ *such that* $\Vert u \Vert _{L^{p}(\Omega_{i})}\leq C_{p}(\Omega_{i}) \Vert u \Vert _{W^{1,q}(\Omega_{i})}$ *for all* $u\in W^{1,q}(\Omega_{i})$. *Then* (1) *holds valid for*

$$C_{p}(\Omega)=M_{p,q}\max_{1\leq i\leq n} C_{p}(\Omega_{i}), $$

*where*

$$\begin{aligned} M_{p,q}= \textstyle\begin{cases} 1&(p\geq q),\\ n^{\frac{1}{p}-\frac{1}{q}}&(p< q). \end{cases}\displaystyle \end{aligned}$$

### Proof

We consider both the cases in which $p<\infty$ and $p=\infty$.

When $p<\infty$, it follows that

$$\begin{aligned} \begin{aligned} \Vert u \Vert _{L^{p}(\Omega)}&= \biggl(\sum _{1\leq i\leq n} \Vert u \Vert _{L^{p}(\Omega_{i})}^{p} \biggr)^{1/p} \\ &\leq \biggl(\sum_{1\leq i\leq n}C_{p}( \Omega_{i})^{p} \Vert u \Vert _{W^{1,q}(\Omega_{i})}^{p} \biggr)^{1/p} \\ &\leq\max_{1\leq i\leq n} C_{p}( \Omega_{i}) \biggl(\sum_{1\leq i\leq n} \Vert u \Vert _{W^{1,q}(\Omega_{i})}^{p} \biggr)^{1/p} \\ &\leq M_{p,q}\max_{1\leq i\leq n} C_{p}( \Omega_{i}) \Vert u \Vert _{W^{1,q}(\Omega)}. \end{aligned} \end{aligned}$$

Note that $\vert x \vert _{p}\leq M_{p,q} \vert x \vert _{q}$ holds for $x=(x_{1},x_{2},\ldots,x_{n})\in\mathbb{R}^{n}$ (see [19, Lemma A.1] for a detailed proof), where we denote

$$\begin{aligned} \vert x \vert _{p}= \textstyle\begin{cases} \displaystyle \biggl(\sum_{1\leq i\leq n} \vert x_{i} \vert ^{p} \biggr)^{\frac{1}{p}}&(1\leq p< \infty),\\ \displaystyle\max_{1\leq i\leq n} \vert x_{i} \vert &(p=\infty). \end{cases}\displaystyle \end{aligned}$$

When $p=\infty$,

$$\begin{aligned} \Vert u \Vert _{L^{\infty}(\Omega)}&= \max_{1\leq i\leq n} \Vert u \Vert _{L^{\infty}(\Omega_{i})} \\ &\leq\max_{1\leq i\leq n} C_{p}( \Omega_{i}) \Vert u \Vert _{W^{1,q}(\Omega_{i})} \\ &\leq\max_{1\leq i\leq n} C_{p}( \Omega_{i})\max_{1\leq i\leq n} \Vert u \Vert _{W^{1,q}(\Omega_{i})}. \end{aligned}$$

Since $M_{\infty,q}=1$, we have

$$\begin{aligned} \Vert u \Vert _{L^{\infty}(\Omega)}&\leq\max_{1\leq i\leq n} C_{p}(\Omega_{i}) \Vert u \Vert _{W^{1,q}(\Omega)}. \end{aligned}$$

 □

## Appendix 2: A proof of Lemma 3.1

This section provides a proof of Lemma 3.1 based on [31, Lemma 7.16].

### Proof of Lemma 3.1

Since $C^{\infty}(\Omega)\cap W^{1,1}(\Omega)$ is densely defined in $W^{1,1}(\Omega)$, it suffices to prove Lemma 3.1 for $u\in C^{1}(\Omega)$. Since Ω is convex, we have, for $x, y\in\Omega$,

$$\begin{aligned} u(x)-u(y)=- \int_{0}^{ \vert x-y \vert }\partial _{r}u(x+r\omega) \,dr, \end{aligned}$$

where $\omega=(y-x)/ \vert y-x \vert $ and $\partial _{r}u(x+r\omega)=\frac{\partial}{\partial r}u(x+r\omega)$. Integrating with respect to *y* over Ω, we obtain

$$\begin{aligned} \bigl\vert u(x)-u_{\Omega}(x) \bigr\vert &= \vert \Omega \vert ^{-1} \biggl\vert \int_{\Omega} \int_{0}^{\vert x-y\vert }\partial_{r}u(x+r\omega)\,dr \,dy \biggr\vert \\ &\leq \vert \Omega \vert ^{-1} \int_{\Omega} \int _{0}^{ \vert x-y \vert } \bigl\vert \partial_{r}u(x+r \omega ) \bigr\vert \,dr\,dy \\ &\leq \vert \Omega \vert ^{-1} \int_{\Omega} \int _{0}^{\infty} \bigl\vert (E_{\Omega,\mathbb{R}^{N}} \partial _{r}u) (x+r\omega) \bigr\vert \,dr\,dy \\ &\leq \vert \Omega \vert ^{-1} \int_{B(x,d_{\Omega})} \int _{0}^{\infty} \bigl\vert (E_{\Omega,\mathbb{R}^{N}} \partial _{r}u) (x+r\omega) \bigr\vert \,dr\,dy \\ &= \vert \Omega \vert ^{-1} \int_{0}^{d_{\Omega}} \int _{ \vert \omega \vert =1} \int_{0}^{\infty} \bigl\vert (E_{\Omega,\mathbb{R}^{N}} \partial_{r}u) (x+r\omega) \bigr\vert \rho ^{N-1}\,dr \,d \omega \,d\rho \\ &= \vert \Omega \vert ^{-1} \int_{0}^{\infty} \int_{ \vert \omega \vert =1} \int_{0}^{d_{\Omega}} \bigl\vert (E_{\Omega,\mathbb{R}^{N}} \partial_{r}u) (x+r\omega) \bigr\vert \rho ^{N-1}\,d\rho \,d \omega \,dr \\ &= \frac{d_{\Omega}^{N}}{N \vert \Omega \vert } \int _{0}^{\infty} \int_{ \vert \omega \vert =1} \bigl\vert (E_{\Omega,\mathbb{R}^{N}}\partial_{r}u) (x+r\omega) \bigr\vert \,d\omega \,dr \\ &= \frac{d_{\Omega}^{N}}{N \vert \Omega \vert } \int_{ \vert \omega \vert =1} \int_{0}^{\infty} \bigl\vert (E_{\Omega ,\mathbb{R}^{N}} \partial_{r}u) (x+r\omega) \bigr\vert r^{1-N}r^{N-1}drd \omega \\ &= \frac{d_{\Omega}^{N}}{N \vert \Omega \vert } \int _{\mathbb{R}^{N}} \bigl\vert (E_{\Omega,\mathbb{R}^{N}}\partial _{r}u) (y) \bigr\vert \vert x-y \vert ^{1-N}\,dy \\ &= \frac{d_{\Omega}^{N}}{N \vert \Omega \vert } \int _{\Omega} \bigl\vert \partial_{r}u(y) \bigr\vert \vert x-y \vert ^{1-N}\,dy. \end{aligned}$$

Therefore, a proof of Lemma 3.1 is completed. □
